# Physician adherence and patient-reported outcomes in heart failure with reduced ejection fraction in the era of angiotensin receptor-neprilysin inhibitor therapy

**DOI:** 10.1038/s41598-022-11740-5

**Published:** 2022-05-11

**Authors:** In-Cheol Kim, Jong-Chan Youn, Se Yong Jang, Sang Eun Lee, Hyun-Jai Cho, Jin-Oh Choi, Ju-Hee Lee, Kyung-Hee Kim, Sun Hwa Lee, Kye Hun Kim, Jong Min Lee, Byung-Su Yoo, the SPARK study group, Byung-Su Yoo, Se Yong Jang, Jong Min Lee, In-Cheol Kim, Jin-Oh Choi, Hyun-Jai Cho, Sang Eun Lee, Kyung-Hee Kim, Kye Hun Kim, Sun Hwa Lee, Ju-Hee Lee, Jung Hyun Choi, Jaewon Oh, Suk Min Seo, Jin Joo Park, Jeong Su Kim, Seok-Jae Hwang, Jae-Hyeong Park, Sang Min Park, Eung Ju Kim, Jong-Chan Youn, Sang-Hyun Ihm, Sang Jin Ha, Wook-Jin Chung, Seong Hoon Choi, Ji-Hyun Kim, Song-Yi Kim, Kyoung Im Cho, Dong Ryeol Ryu

**Affiliations:** 1 Division of Cardiology, Department of Internal Medicine, Keimyung University Dongsan Hospital, Keimyung University College of Medicine, Daegu, Republic of Korea; 2 Division of Cardiology, Department of Internal Medicine, Seoul St. Mary’s Hospital, Catholic Research Institute for Intractable Cardiovascular Disease, College of Medicine, The Catholic University of Korea, Seoul, Republic of Korea; 3 Division of Cardiology, Department of Internal Medicine, Kyungpook National University Hospital, Daegu, Republic of Korea; 4 Division of Cardiology, Department of Internal Medicine, Asan Medical Center, Seoul, Republic of Korea; 5 Division of Cardiology, Department of Internal Medicine, Seoul National University Hospital, Seoul, Republic of Korea; 6 Division of Cardiology, Department of Internal Medicine, Samsung Medical Center, Seoul, Republic of Korea; 7 Division of Cardiology, Department of Internal Medicine, Chungbuk National University Hospital, Cheongju, Republic of Korea; 8 Division of Cardiology, Department of Internal Medicine, Incheon Sejong Hospital, Incheon, Republic of Korea; 9 Division of Cardiology, Department of Internal Medicine, Jeonbuk National University Hospital, Jeonju, Republic of Korea; 10 Division of Cardiology, Department of Internal Medicine, College of Medicine, Chonnam National University Hospital, Gwangju, Republic of Korea; 11 Division of Cardiology, Department of Internal Medicine, Uijeongbu St. Mary’s Hospital, College of Medicine, The Catholic University of Korea, Uijeongbu, Republic of Korea; 12 Division of Cardiology, Department of Internal Medicine, Wonju Severance Christian Hospital, Yonsei University Wonju College of Medicine, 20 Ilsan-ro, 26426 Wonju, Gangwon-do, Republic of Korea; 13 Division of Cardiology, Department of Internal Medicine, Pusan National University Hospital, Busan, Republic of Korea; 14 Division of Cardiology, Department of Internal Medicine, Severance Hospital, Yonsei University College of Medicine, Seoul, Republic of Korea; 15 Division of Cardiology, Department of Internal Medicine, Seoul St. Mary’s Hospital, The Catholic University of Korea, Seoul, Republic of Korea; 16 Division of Cardiology, Department of Internal Medicine, Seoul National University College of Medicine, Seoul National University Bundang Hospital, Seoul, Republic of Korea; 17 Division of Cardiology, Department of Internal Medicine, Pusan National University Yangsan Hospital, Busan, Republic of Korea; 18 Division of Cardiology, Department of Internal Medicine, Gyeongsang National University Hospital, Jinju, Republic of Korea; 19 Division of Cardiology, Department of Internal Medicine, School of Medicine, Chungnam National University, Chungnam National University Hospital, Daejeon, Republic of Korea; 20 Division of Cardiology, Department of Internal Medicine, Chuncheon Sacred Heart Hospital, Hallym University College of Medicine, Seoul, Republic of Korea; 21 Division of Cardiology, Cardiovascular Center, Korea University Guro Hospital, Korea University College of Medicine, Seoul, Republic of Korea; 22 Department of Internal Medicine, College of Medicine, The Catholic University of Korea, Bucheon St. Mary’s Hospital, Bucheon, Republic of Korea; 23 Division of Cardiology, Department of Internal Medicine, Gangneung Asan Hospital, University of Ulsan College of Medicine, Seoul, Republic of Korea; 24 Division of Cardiology, Department of Internal Medicine, Gachon Cardiovascular Research Institute, Gachon University Gil Medical Center, Incheon, Republic of Korea; 25 Division of Cardiology, Department of Internal Medicine, Kangnam Sacred Heart Hospital, Hallym University College of Medicine, Seoul, Republic of Korea; 26 Division of Cardiology, Department of Internal Medicine, Cardiovascular Center, Dongguk University Ilsan Hospital, Dongguk, Republic of Korea; 27 Deparment of Cardiology, Jeju National University Hospital, Jeju National University School of Medicine, Jeju, Republic of Korea; 28 Division of Cardiology, Department of Internal Medicine, Kosin University Gospel Hospital, Kosin University College of Medicine, Busan, Republic of Korea; 29 Division of Cardiology, Department of Internal Medicine, Kangwon National University Hospital, Kangwon National University, School of Medicine, Seoul, Republic of Korea

**Keywords:** Cardiology, Outcomes research

## Abstract

This Korean nationwide, multicenter, noninterventional, prospective cohort study aimed to analyze physician adherence to guideline-recommended therapy for heart failure (HF) with reduced ejection fraction (HFrEF) and its effect on patient-reported outcomes (PROs). Patients diagnosed with or hospitalized for HFrEF within the previous year were enrolled. Treatment adherence was considered optimal when all 3 categories of guideline-recommended medications (angiotensin-converting enzyme inhibitors, angiotensin receptor blockers, or angiotensin receptor-neprilysin inhibitors; beta-blockers; and mineralocorticoid receptor antagonists) were prescribed and suboptimal when ≤ 2 categories were prescribed. The 36-Item Short Form Survey (SF-36) scores were compared at baseline and 6 months between the 2 groups. Overall, 854 patients from 30 hospitals were included. At baseline, the optimal adherence group comprised 527 patients (61.7%), whereas during follow-up, the optimal and suboptimal adherence groups comprised 462 (54.1%) and 281 (32.9%) patients, respectively. Patients in the suboptimal adherence group were older, with a lower body mass index, and increased comorbidities, including renal dysfunction. SF-36 scores were significantly higher in the optimal adherence group for most domains (*P* < 0.05). This study showed satisfactory physician adherence to contemporary treatment for HFrEF. Optimal adherence to HF medication significantly correlated with better PROs.

## Introduction

Heart failure (HF) is a major threat globally, owing to its association with increased morbidity, mortality, and deterioration in the quality of life of patients. It also imposes a substantial economic burden on the society^1–3^. Recent HF guidelines recommend evidence-based medications to reduce mortality in patients with HF with reduced ejection fraction (HFrEF)^4^. These medications include angiotensin-converting enzyme inhibitors (ACEis)/angiotensin receptor blockers (ARBs), beta-blockers (BBs), and mineralocorticoid receptor antagonists (MRAs)^1–3,5^. Based on the results of landmark randomized controlled trials, angiotensin receptor-neprilysin inhibitors (ARNIs) were included as a class I indication in the recently updated HF guidelines. Subsequently, recent trials have investigated the potential of ARNIs in increasing the survival and quality of life of patients with HF^6–9^. However, to date, large-scale performance-measure trials in HF have not included patients treated with ARNIs, a drug class that can improve patient outcomes and treatment cost-effectiveness^8,10^. The changing clinical profiles of patients with HFrEF necessitate a reassessment of patient quality of life and physician adherence to the latest HF guidelines, which now include 5 different classes of recommended medications (class I indication).

Moreover, a gap exists between the recommended guidelines and the current practice of HF treatment with respect to physician adherence. An observational nationwide study using the Korean National Health Insurance Claims database showed that 28.6% of elderly patients with HF did not receive optimal evidence-based treatment^11^. Furthermore, compelling data from a previous study show that many patients with HF do not take their medications as prescribed by their healthcare providers. According to these data, the overall compliance among patients with HF was 72%, and noncompliance contributed to worsening of HF symptoms, thereby leading to hospitalization^12^. Therefore, further studies on contemporary medical treatment patterns, including use of ARNIs, in patients with HFrEF are necessary to gain insights into improved treatment strategies in these patients.

Poor quality of life resulting from uncontrolled symptoms is a major concern and an important target for treatment in patients with HF. Notably, patients with HF with uncontrolled symptoms had low scores in all domains of the 36-Item Short Form Survey (SF-36), a tool designed to evaluate quality-of-life parameters such as physical health and mental status^13^. Patient-reported outcome (PRO) measures are used to evaluate patient-reported information in terms of subjective health, including health-related quality of life, symptoms of anxiety and depression, and symptom burden. Evaluation of PROs in patients with HFrEF is important because HF is a common chronic illness that is accompanied by frequent acute exacerbations, often requiring hospitalization, and a significant symptom burden^14^. PROs enable physicians to deliver more patient-centered care, thereby improving patients’ quality of life. A recent study showed that nonadherence to medication also correlated with poor PROs^15^. Real-world nationwide data on adherence to guideline-recommended treatment and PROs in Asian patients with HF are lacking with respect to ARNIs. Therefore, this study aimed to analyze the treatment patterns of patients with HFrEF in the era of ARNIs and to evaluate PROs according to physician adherence to guideline-recommended treatments.

## Methods

### Study design and patient population

**Figure 1:**
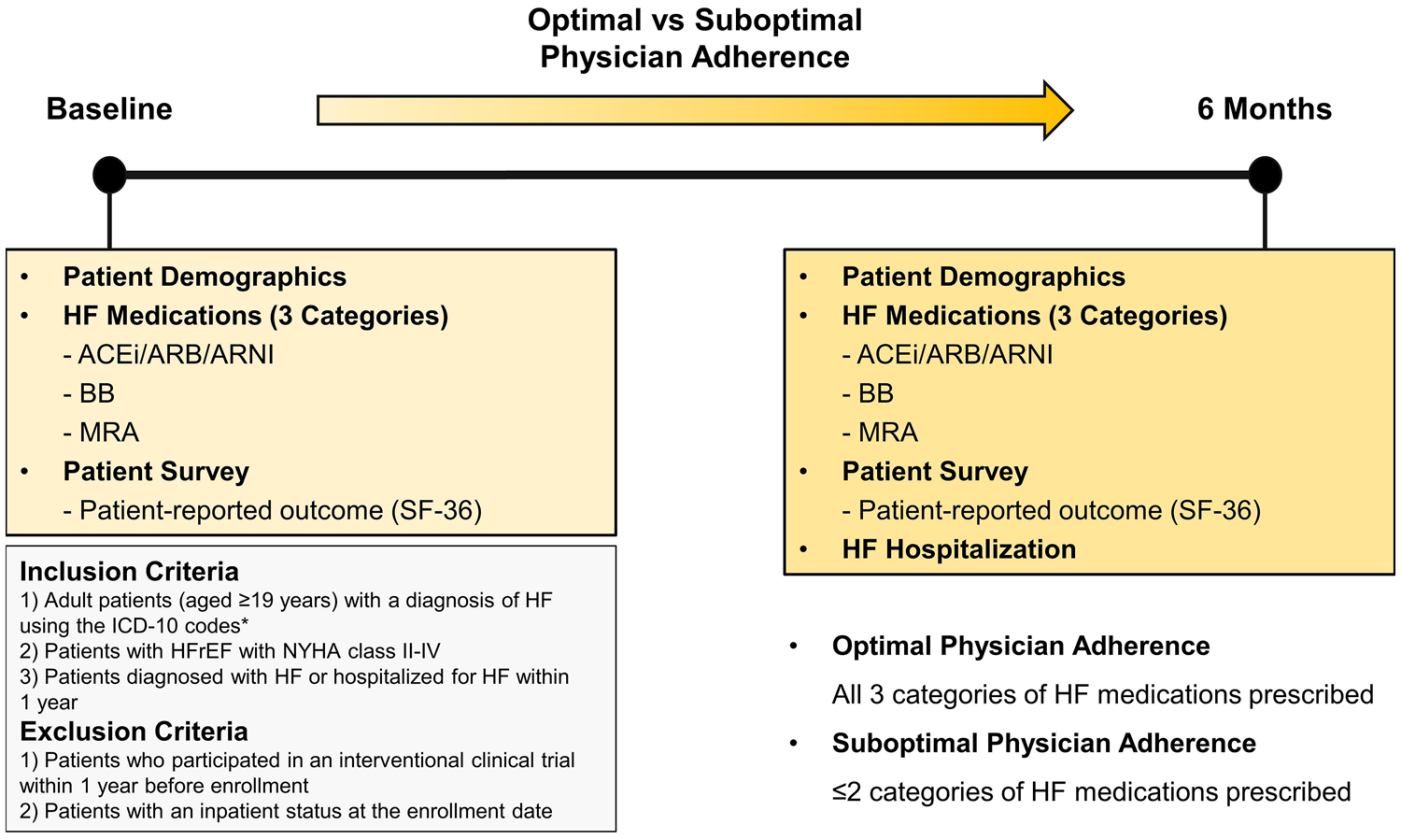
Schematic summary of the study design. ACEi, angiotensin-converting enzyme inhibitor; ARB, angiotensin receptor blocker; ARNI, angiotensin receptor-neprilysin inhibitor; BB, beta-blocker; HF, heart failure; HFrEF, heart failure with reduced ejection fraction; ICD-10, International Classification of Diseases 10th Revision; MRA, mineralocorticoid receptor antagonist; NYHA, New York Heart Association; SF-36, 36-Item Short Form Survey. *I50, I50.0, I50.1, I50.9, I11.0, I13.0, I13.2, I25.5, I42, I42.x.

This was a Korean nationwide, multicenter, noninterventional, prospective cohort study. Patients with HFrEF who were diagnosed with HF or hospitalized for HF within the previous year were enrolled on an outpatient basis. Data were collected at enrollment (baseline) and after 6 months by review of medical records and patient paper surveys. Only patients who completed the 6-month follow-up were included in the final analysis. Adult patients aged ≥ 19 years with a documented diagnosis of HF using the International Classification of Diseases 10th Revision (ICD-10) codes I50, I50.0, I50.1, I50.9, I11.0, I13.0, I13.2, I25.5, I42, or I42.x on the main or first subdiagnosis; patients with HFrEF with a left ventricular ejection fraction (LVEF) < 40% and New York Heart Association class II-IV; patients diagnosed with HF or hospitalized for HF within the previous year; and patients with an outpatient status at the enrollment date were included in the analysis. Patients who had participated in an interventional clinical trial 1 year before enrollment and patients with an inpatient status at the enrollment date were excluded. The study design is summarized in Fig. 1.

### Ethics statement

The study was performed in accordance with the principles outlined in the Declaration of Helsinki and was approved by the institutional review board of Wonju Severance Christian Hospital (IRB number CR317108) and each participating center (Asan Medical Center; Bucheon St. Mary’s Hospital; Chonnam National University Hospital; Chungbuk National University Hospital; Chungnam National University Hospital; Dongguk University Ilsan Hospital; Gachon University Gil Medical Center; Gangneung Asan Hospital; Gyeongsang National University Hospital; Hallym University Chuncheon Sacred Heart Hospital; Hallym University Dongtan Sacred Heart Hospital; Hallym University Kangnam Sacred Heart Hospital; Incheon Sejong Hospital; Jeju National University Hospital; Jeonbuk National University Hospital; Kangwon National University Hospital; Keimyung University Dongsan Hospital; Korea University Guro Hospital; Kosin University Gospel Hospital; Kyoungpook National University Chilgok Hospital; Kyungpook National University Hospital; Pusan National University Hospital; Pusan National University Yangsan Hospital; Samsung Medical Center; Seoul National University Bundang Hospital; Seoul National University Hospital; Seoul St. Mary's Hospital; Severance Hospital; Uijeongbu St. Mary's Hospital.). All patients provided written informed consent before enrollment in the study.

### Guideline adherence measures

Adherence to guideline-directed medical therapy for HFrEF (3 categories: ACEis/ARBs/ARNIs, BBs, and MRAs) was evaluated using the prescription information at baseline and after a follow-up of 6 months. Adherence to treatment was considered optimal when all 3 categories of medications were prescribed and suboptimal when ≤ 2 categories of medications were prescribed.

### Outcome analysis

The primary objective of the study was to evaluate the treatment patterns of patients with HFrEF according to physician adherence in the era of ARNIs. Patient characteristics and treatment patterns were obtained from hospital medical records.

Quality of life was measured using SF-36^16^, which provides scores for physical health as well as mental status. SF-36 consists of the following 10 domains: physical functioning (PF), role physical (RP), bodily pain (BP), general health (GH), vitality (VT), social functioning (SF), role emotional (RE), mental health (MH), physical component summary (PCS), and mental component summary (MCS). The scores for each domain range from 0 to 100, with higher scores indicating better quality of life.

SF-36 scores were compared between the optimal adherence and suboptimal adherence groups at baseline and at 6 months. In addition, the time to HF hospitalization, defined as the duration from study enrollment to hospitalization for HF during the 6-month follow-up, was compared between the 2 groups.

### Statistical analysis

All data were analyzed without adjustments for outliers or imputation for missing values. Categorical variables are presented as numbers and frequencies, whereas continuous variables are presented as means ± standard deviations. A paired *t*-test was used to compare the variables at baseline and at the 6-month follow-up within each group. Comparisons of continuous variables between the optimal adherence group and suboptimal adherence group were made using the Student’s independent 2-sample *t*-test. For categorical variables, the chi-square test was used to compare the data between the 2 adherence groups. The log-rank test was used to compare time to hospitalization due to HF between the 2 groups. A 2-tailed *P* value of < 0.05 was considered statistically significant. Statistical analyses were performed using IBM SPSS Statistics, version 21 (Armonk, NY, USA).

## Results

### Patient baseline characteristics

A total of 975 patients from 30 hospitals were enrolled, of whom 854 completed the 6-month follow-up and were included in the analysis. Patients were enrolled from July 2018 to April 2019, and data were collected from July 2018 to November 2019, with a mean ± standard deviation follow-up duration of 183.4 ± 28.7 days (Supplementary Figure S1). Of the 854 patients, 527 (61.7%) were in the optimal adherence group and 327 (38.3%) were in the suboptimal adherence group based on the prescription data collected at enrollment (30.4% of the patients were prescribed medications from 2 categories and 7.6% from 1 category; 0.2% of the patients were not prescribed any of the HF medications; Table 1). Patients in the optimal adherence group were younger and predominantly male, had a higher body mass index, and had fewer comorbidities with preserved renal function. During follow-up, 462 (54.1%) patients remained in the optimal adherence group, 65 (7.6%) patients moveed from optimal to suboptimal adherence, 46 (5.4%) patients changed from suboptimal to optimal adherence, and 281 (32.9%) patients remained in the suboptimal adherence group. Patients’ characteristics evaluated at the 6-month follow-up are summarized in Supplementary Table 1.

**Table 1:** Baseline characteristics of patients with optimal adherence and suboptimal adherence at enrollment.

Characteristics	Optimal adherence (n = 527)	Suboptimal adherence (n = 327)	*P* Value^a^
Age (years)	60.2 ± 14.4	65.1 ± 13.5	< 0.001
Sex (male)	377 (71.5)	208 (63.6)	0.015
BMI (kg/m^2^)	24.8 ± 4.7	23.3 ± 3.7	< 0.001
HF duration > 12 months	135 (25.6)	97 (29.7)	0.196
**NYHA class**			
Class II	437 (82.9)	257 (78.6)	0.218
Class III	86 (16.3)	65 (19.9)	
Class IV	4 (0.8)	5 (1.5)	
LVEF (%)	28.1 ± 7.0	29.7 ± 6.7	0.001
BNP (pg/mL)	971.5 ± 1328.1	1110.6 ± 1338.0	0.453
NT-proBNP (pg/mL)	3272.8 ± 4873.4	7125.9 ± 9,770.3	< 0.001
Hb (g/dL)	13.8 ± 2.1	12.8 ± 2.4	< 0.001
Na (mmol/L)	139.4 ± 3.0	139.5 ± 3.3	0.509
Cl (mmol/L)	102.3 ± 3.8	103.2 ± 4.6	0.007
K (mmol/L)	4.5 ± 0.5	4.4 ± 0.5	0.189
BUN (mg/dL)	20.9 ± 10.3	26.6 ± 16.2	< 0.001
Cr (mg/dL)	1.2 ± 0.9	1.7 ± 1.8	< 0.001
CCr (mL/min)	77.2 ± 38.9	57.6 ± 41.3	< 0.001
**Comorbidities (yes)**	435 (82.5)	292 (89.3)	0.007
Hypertension	254 (48.2)	187 (57.2)	0.011
Atrial fibrillation	129 (24.5)	100 (30.6)	0.050
Dyslipidemia	189 (35.9)	132 (40.4)	0.187
Diabetes mellitus	175 (33.2)	129 (39.5)	0.064
COPD	29 (5.5)	28 (8.6)	0.082
MI	71 (13.5)	52 (15.9)	0.326
PCI	102 (19.4)	76 (23.2)	0.174
CABG	17 (3.2)	16 (4.9)	0.219
ESRD	8 (1.5)	24 (7.3)	< 0.001
Number of comorbidities	1.9 ± 1.4	2.3 ± 1.5	< 0.001

Data are presented as number (%) or mean ± standard deviation.

BMI, body mass index; BNP, B-type natriuretic peptide; BUN, blood urea nitrogen; CABG, coronary artery bypass grafting; CCr, creatinine clearance; Cl, chlorine; COPD, chronic obstructive pulmonary disease; Cr, creatinine; ESRD, end-stage renal disease; Hb, hemoglobin; HF, heart failure; K, potassium; LVEF, left ventricular ejection fraction; MI, myocardial infarction; Na, sodium; NT-proBNP, N-terminal pro B-type natriuretic peptide; NYHA, New York Heart Association; PCI, percutaneous coronary intervention.

^a^*P* values were calculated using the Student’s independent 2-sample *t*-test or χ^2^ test as appropriate.

### Treatment patterns in patients with HFrEF

**Figure 2:**
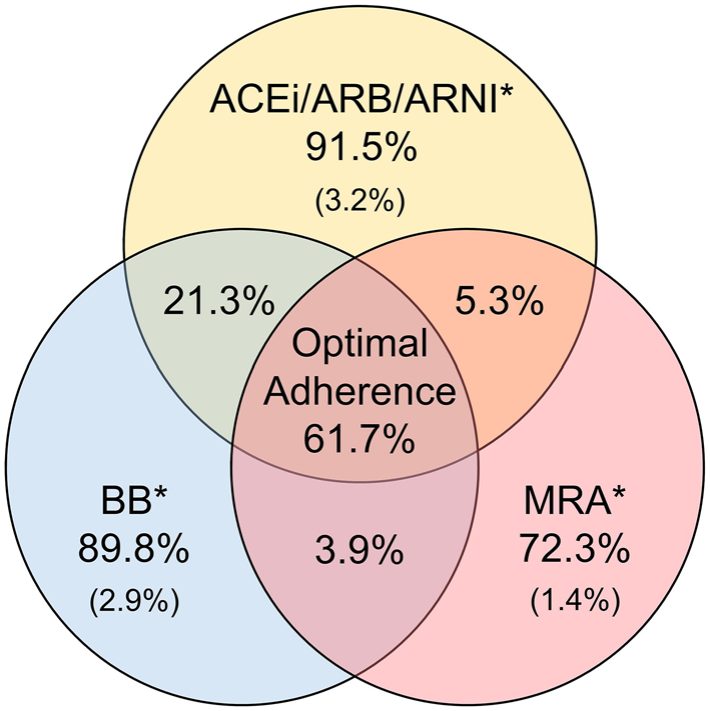
Treatment patterns of guideline-recommended medications at baseline. ACEi, angiotensin-converting enzyme inhibitor; ARB, angiotensin receptor blocker; ARNI, angiotensin receptor-neprilysin inhibitor; BB, beta-blocker; HF, heart failure; MRA, mineralocorticoid receptor antagonist. *Indicates the percentage of patients who were prescribed only 1 category of HF medication.

Overall, 91.5%, 89.8%, and 72.3% of patients were prescribed ACEis/ARBs/ARNIs (19.3%/39.9%/32.4%), BBs, and MRAs, respectively (Fig. 2). At the 6-month follow-up, 91.3%, 89.2%, and 68.0% of patients were prescribed ACEis/ARBs/ARNIs (11.4%/46.5%/33.5%), BBs, and MRAs, respectively. Among ACEis, perindopril was most frequently prescribed (58.8%), followed by ramipril (35.1%), whereas among ARBs, valsartan was most frequently prescribed (46.5%), followed by candesartan (32.2%). Among BBs, carvedilol was most frequently prescribed (56.2%), followed by bisoprolol (31.9%). Only 2 patients (0.2%) were not prescribed any of the guideline-recommended medications.

### PRO measures

**Figure 3:**
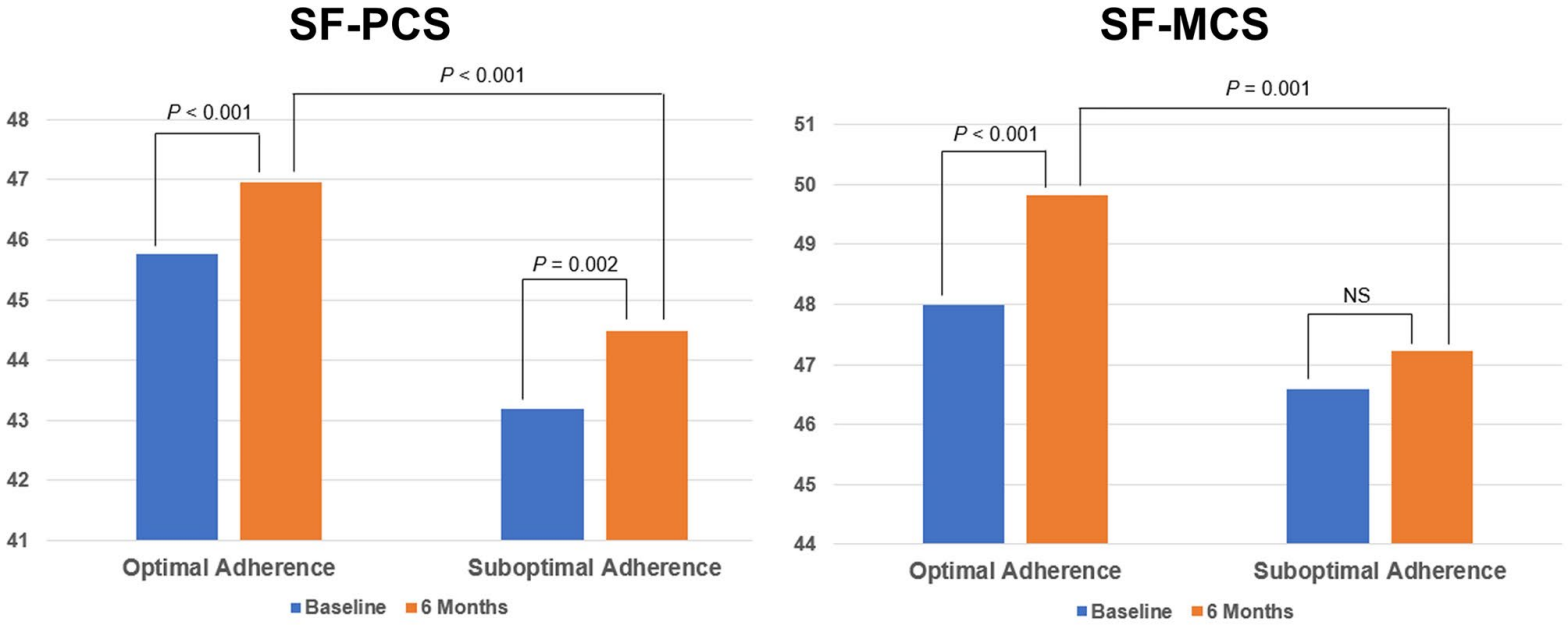
Comparison of SF-36 scores according to physician adherence at baseline and at the 6-month follow-up. MCS, mental component summary; NS, not significant; PCS, physical component summary; SF-36, 36-Item Short Form Survey.

SF-36 scores for PROs at baseline and at the 6-month follow-up were significantly better in the optimal adherence group for most domains. The optimal adherence group had significantly higher scores at baseline for all domains, except VT, MH, and MCS, than the suboptimal adherence group (PF, *P* < 0.001; RP, *P* = 0.002; BP, *P* = 0.037; GH, *P* = 0.006; VT, *P* = 0.050; SF, *P* = 0.002; RE, *P* = 0.002; MH, *P* = 0.275; PCS, *P* < 0.001; and MCS, *P* = 0.081). At the 6-month follow-up, SF-36 scores for all domains were significantly higher in the optimal adherence group than in the suboptimal adherence group (PF, *P* < 0.001; RP, *P* < 0.001; BP, *P* = 0.029; GH, *P* = 0.001; VT, *P* = 0.002; SF, *P* = 0.001; RE, *P* < 0.001; MH, *P* = 0.001; PCS, *P* < 0.001; and MCS, *P* = 0.001). A significant increase in scores during the follow-up period was also noted in the optimal adherence group, whereas the increase in scores was less prominent in the suboptimal adherence group. SF-36 scores are presented in Table 2 and Fig. 3.

**Table 2:** Comparison of SF-36 scores between the optimal adherence and suboptimal adherence groups at baseline and at the 6-month follow-up.

SF-36								
Domain	Baseline			6 Months			*P* Value^b^ Within the Optimal Adherence Group	*P* Value^c^ Within the Suboptimal Adherence Group
	Optimal adherence (n = 527)	Suboptimal adherence (n = 327)	*P* Value^a^	Optimal adherence (n = 503)	Suboptimal adherence (n = 314)	*P* Value^a^		
PF	64.9 ± 26.7	56.4 ± 30.0	< 0.001	67.3 ± 26.8	58.7 ± 29.8	< 0.001	0.003	0.117
RP	64.1 ± 31.3	56.9 ± 33.5	0.002	70.9 ± 29.4	62.1 ± 34.0	< 0.001	< 0.001	0.002
BP	74.8 ± 27.3	70.6 ± 28.8	0.037	79.1 ± 25.1	75.0 ± 27.2	0.029	< 0.001	0.012
GH	53.3 ± 19.0	49.4 ± 20.6	0.006	55.3 ± 18.5	50.5 ± 20.5	0.001	0.022	0.324
VT	50.6 ± 22.3	47.5 ± 23.0	0.050	51.8 ± 21.2	46.8 ± 23.0	0.002	0.215	0.351
SF	76.1 ± 26.6	69.9 ± 30.2	0.002	80.9 ± 25.0	74.1 ± 31.0	0.001	< 0.001	0.010
RE	75.8 ± 28.3	69.1 ± 32.6	0.002	81.5 ± 25.5	73.6 ± 31.0	< 0.001	< 0.001	0.012
MH	69.6 ± 20.6	67.9 ± 22.1	0.275	72.9 ± 18.0	68.1 ± 22.2	0.001	< 0.001	0.726
PCS	45.8 ± 9.1	43.2 ± 9.8	< 0.001	47.0 ± 9.0	44.5 ± 9.8	< 0.001	< 0.001	0.002
MCS	48.0 ± 10.6	46.6 ± 11.7	0.081	49.8 ± 9.1	47.2 ± 11.5	0.001	< 0.001	0.424

Data are presented as number (%) or mean ± standard deviation.

BP, bodily pain; GH, general health; MCS, mental component summary; MH, mental health; PCS, physical component summary; PF, physical functioning; RE, role emotional; RP, role physical; SF, social functioning; SF-36, 36-Item Short Form Health Survey; VT, vitality.

^a^*P* values for the difference between the adherence groups were calculated using the Student’s independent 2-sample *t*-test.

^b^*P* values for the difference within the optimal adherence group were calculated using a paired *t*-test.

^c^*P* values for the difference within the suboptimal adherence group were calculated using a paired *t*-test.

### Time to HF hospitalization

A total of 49 patients (9.3%) in the optimal adherence group and 34 patients (10.4%) in the suboptimal adherence group were hospitalized for HF during the 6-month follow-up. The log-rank test showed no significant difference between the 2 groups (*P* = 0.549).

## Discussion

This nationwide, multicenter, prospective cohort study demonstrated that (1) physician adherence to guideline-directed medical therapy was satisfactory (92.1% of patients were prescribed > 2 categories of HFrEF medications, and 61.7% of patients were prescribed all 3 categories); (2) patients in the suboptimal adherence group tended to be older and had a lower body mass index and increased comorbidities, including renal dysfunction; and (3) patients in the optimal adherence group had better PROs at baseline and a more prominent improvement in PROs at the 6-month follow-up compared with those in the suboptimal adherence group.

### Treatment patterns in patients with HFrEF

**Figure 4:**
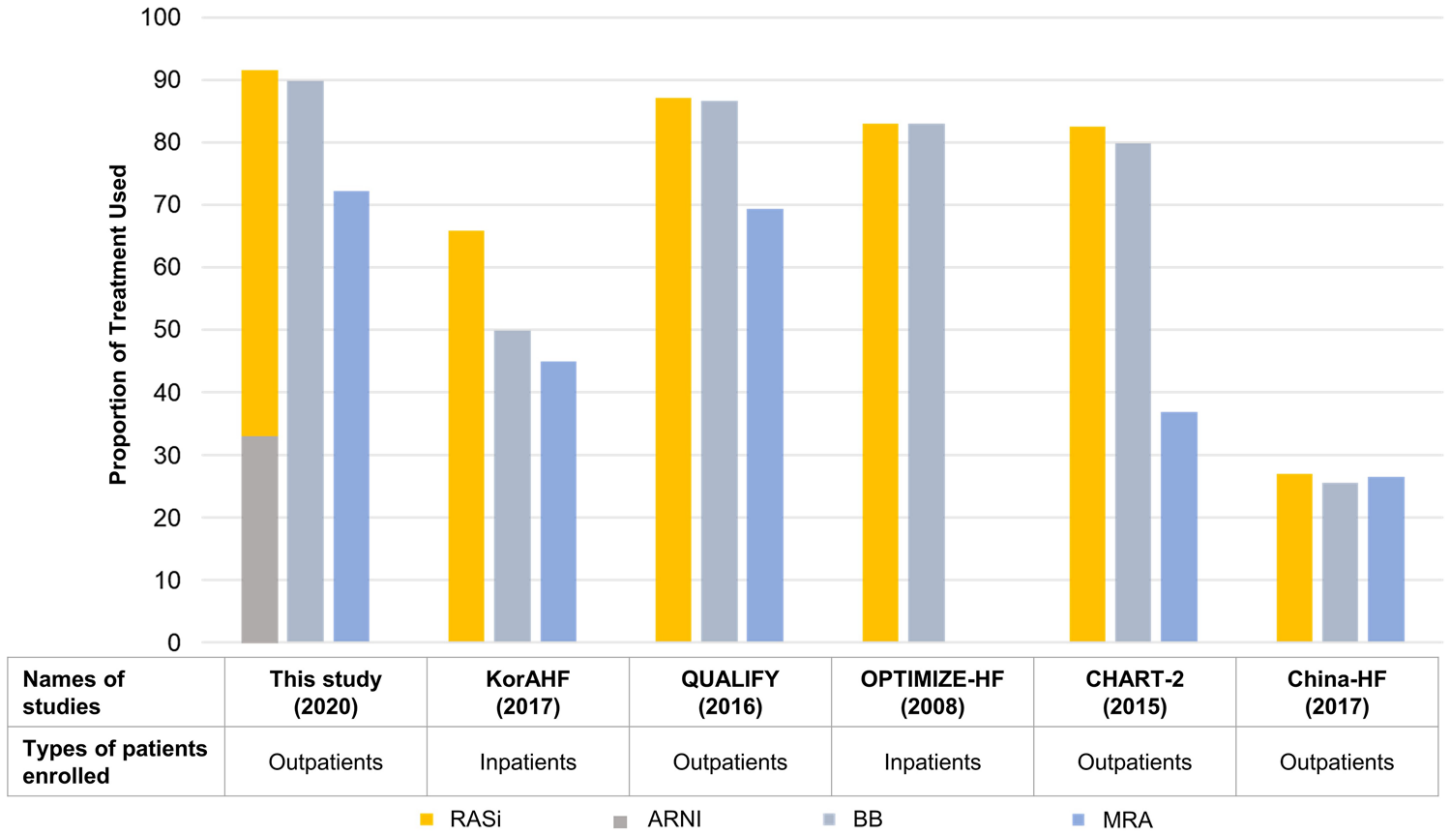
Comparison of physician adherence in this study with previous heart failure registry studies. ARNI, angiotensin receptor-neprilysin inhibitor; BB, beta-blocker; MRA, mineralocorticoid receptor antagonist; RASi, renin–angiotensin–aldosterone system inhibitor.

Data regarding treatment patterns obtained from the current Korean nationwide prospective cohort study are more robust compared with the data obtained from previous international or national registries^17–21^. Data on physician adherence from previous HF registries or trials were focused only on the 3 categories of guideline-recommended HF medications—ACEis/ARBs/ARNIs, BBs, and MRAs. Data from these HF registries^17–21^ revealed a lower prescription rate for all 3 categories of medications. However, recent prospective HFrEF trials showed a higher prescription rate for these medications^15^. In the present study, the use of renin-angiotensin system blockers was 91.5%, which included ARNIs (32.4%). The use of BBs and MRAs was also high, accounting for 89.8% and 72.3%, respectively. Compared with previous registry data in the country, the current data show a substantial improvement in physician adherence to HF therapy guidelines, particularly with respect to the use of BBs (49.9% in the The Korean Acute Heart Failure (KorAHF); Fig. 4)^17^. Compared with previous studies, this study showed a satisfactory prescription rate, which could be attributed to the effect of indirect intervention that may have enhanced physician awareness, and the fact that most patients in this study were enrolled at tertiary university hospitals with dedicated HF specialists. Moreover, the emergence of ARNI, a new treatment option for HFrEF, and its large diffusion could have also influenced adherence to HF therapy guidelines.

### Physician adherence and PROs in HF

Patient adherence to guideline-directed HF medications is crucial to reduce mortality and morbidity^1–3,22^. Physician adherence is also important in the management of patients with HF^20,23–27^. PRO measures indicative of feelings or functions related to the patient’s health and treatments are considered important indicators of HF, owing to the significant symptom burden of the disease^14^. Among the various PRO measures, SF-36 provides information on a wide range of parameters indicative of health status. SF-36 has been validated for measuring health-related quality of life in patients with chronic illnesses in several countries^28–31^, but data on the Asian population are still lacking. The results of this study showed that optimal adherence was associated with higher SF-36 scores at baseline, and a noticeable improvement was observed at the 6-month follow-up. However, considerable improvements were also observed in several SF-36 domains in the suboptimal adherence group as patients in this group received at least some degree of guideline-directed pharmacological treatment, and nonpharmacological treatment was administered regardless of the medication status during the study period. SF-36 scores observed in this study were higher than those reported in the previous registry data from Western countries^32^; this may be attributable to the prospective nature of the current study, which might have had an indirect effect on physician adherence, resulting in higher PRO scores.

### Patient adherence to medication in HF

Various measures have been developed and applied to increase patient-level adherence or compliance for better outcomes^33^. The interventions implemented in the current study to improve patient adherence, such as training/education sessions, patient reminder systems, self-care support, physician factors, and organizational changes, were effective in decreasing mortality and rehospitalization.

In the previous study, patients’ noncompliance to therapy was significantly associated with an increase in the number of medicines^34^. This result indicates the difficulty in maintaining optimal compliance with an increase in the number of medications. Physicians need to pay continuous attention during their everyday practice to maintain an optimal level of treatment in patients with HF.

### Variables related to suboptimal physician adherence

According to a previous study, the factors associated with underuse of guideline-directed therapy in HF were female sex, older age, renal dysfunction, liver dysfunction, bradycardia, and hypotension^35^. These factors also correlated with poor outcome and further increased the risk of poor physician adherence to optimal management^36^. In this study, we observed that older age, female sex, low body mass index, more diverse comorbidities, elevated NT-proBNP, decreased renal function, and a relatively preserved left ventricular systolic function were consistently associated with suboptimal adherence at baseline and at the 6-month follow-up. Worsening renal function and combined comorbidities associated with polypharmacy may interfere with optimal HFrEF medication in these patients. Significantly higher NT-proBNP level also reflect the high-risk features of suboptimal adherence group. However, patients with comorbidities who are at higher risk of adverse outcomes require more intensive optimal medical therapy. Patients with relatively preserved left ventricular systolic function (LVEF > 35% to < 40%) are not frequently prescribed all 3 categories of medications as the current guidelines do not recommend routine use of MRAs for LVEF > 35% and partly because of physician inertia in patients with a relatively higher LVEF. Improving physician adherence is crucial, particularly in the context of such high-risk patients. As the aging population increases worldwide, particularly in Asia (including Korea), the issue of adherence trend linked to underlying patient factors needs to be addressed.

### Strengths and limitations

To the best of our knowledge, this is the first prospective cohort study to evaluate the effect of physician adherence on PROs in the era of ARNIs. Current HF guidelines recommend the use of ARNIs over ACEis/ARBs to improve outcomes. Considerable use of ARNIs (> 32%) is reflective of the current real-world treatment pattern for HF.

Despite the above-mentioned strengths, this study has several potential limitations. First, adherence to guidelines was evaluated by medication categories without considering the target dose. However, because of the diversity in the up-titration and dose-adjustment process, calculation of the absolute value of the medication dose during a specific period is difficult. Furthermore, a substantial number of patients cannot reach the target doses even in prospective trials, and a lower dose of medication has proven to be effective in those trials. Second, unlike the previous studies on physician adherence, this study showed no difference in clinical events (HF hospitalizations). This could be due to the short follow-up period and the relatively lower incidence of adverse events observed in the present study. The nonblinded nature of the study might have had an impact on physician and patient awareness, resulting in better HF education and self-management in the entire study population, thereby weakening the early effect of optimal physician adherence. Third, the patients included in this study were relatively younger than those in the previous HF registry study. The trend toward a younger study population has also been seen in previous PRO studies that required patients to have preserved cognitive function to answer the survey questions. Extended observation might elucidate the effect of physicians’ optimal adherence on clinical outcomes.

## Conclusions

Data from the Korean nationwide, prospective registry showed favorable physician adherence to contemporary medical therapy for HFrEF. Optimal physician adherence to HF medications was associated with better PROs. Further studies are required to elucidate the effect of optimal physician adherence on clinical outcomes.

## Supplementary Information

Supplementary Figure 1.

Supplementary Information.

## Data Availability

The data that support the findings of this study are available from Novartis Korea Ltd. but restrictions apply to the availability of these data, which were used under license for the current study, and so are not publicly available. Data are however available from the authors upon reasonable request and with permission of Novartis Korea Ltd.
