# Supplementary figures and images for: Physician adherence and patient-reported outcomes in heart failure with reduced ejection fraction in the era of angiotensin receptor-neprilysin inhibitor therapy
Source: Sci Rep. 2022 May 11;12:7730. doi: 10.1038/s41598-022-11740-5 (PMC9095619; doi:10.1038/s41598-022-11740-5)

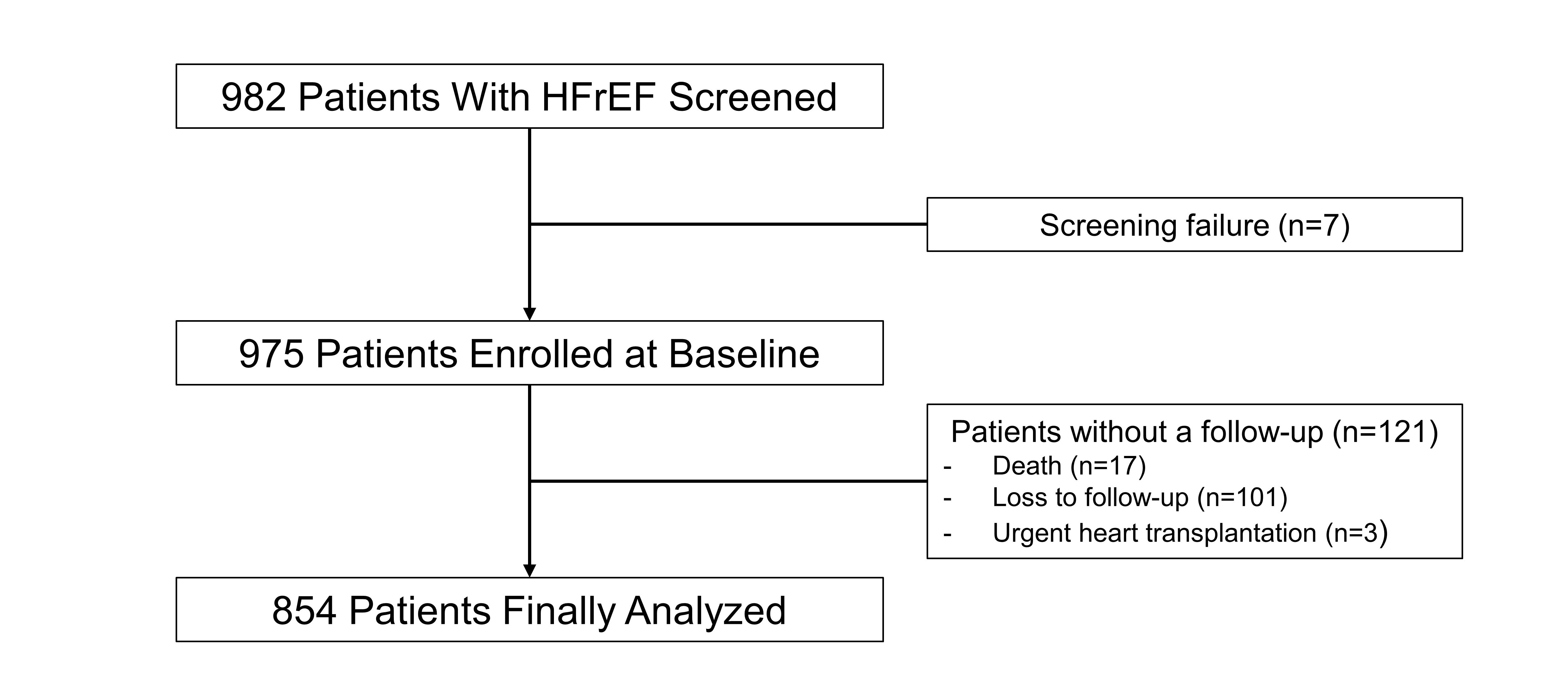

Supplement: Supplementary file 1 — Supplementary Figure 1. [file 41598_2022_11740_MOESM1_ESM.jpg]
