# Supplementary material for: Physician adherence and patient-reported outcomes in heart failure with reduced ejection fraction in the era of angiotensin receptor-neprilysin inhibitor therapy
Source: Sci Rep. 2022 May 11;12:7730. doi: 10.1038/s41598-022-11740-5 (PMC9095619; doi:10.1038/s41598-022-11740-5)
Supplement: Supplementary file 2 — Supplementary Information. [file 41598_2022_11740_MOESM2_ESM.docx]

**Supplementary Figure 1. Flowchart of Patient Population Included in the Study**

HFrEF, heart failure with reduced ejection fraction.

**Supplementary Table 1. Characteristics of Patients in the Optimal Adherence and Suboptimal Adherence Groups at the 6-Month Follow-up**

| **Characteristics** | **Optimal Adherence**  **(n = 508)** | **Suboptimal Adherence**  **(n = 346)** | ***P* Value** ^a^ |
| --- | --- | --- | --- |
| Age (years) | 60.3 ± 14.5 | 64.7 ± 13.6 | < 0.001 |
| Gender (male) | 352 (69.3) | 233 (67.3) | 0.547 |
| BMI (kg/m^2^) | 24.8 ± 4.7 | 23.4 ± 3.7 | < 0.001 |
| HF duration >12 months | 130 (25.6) | 102 (29.5) | 0.210 |
| NYHA class |  |  |  |
| Class II | 423 (83.3) | 271 (78.3) | 0.165 |
| Class III | 81 (15.9) | 70 (20.2) |  |
| Class IV | 4 (0.8) | 5 (1.5) |  |
| LVEF (%) | 27.9 ± 6.9 | 29.9 ± 6.8 | < 0.001 |
| BNP (pg/mL) | 1004.5 ± 1360.4 | 1054.6 ± 1298.9 | 0.784 |
| NT-proBNP (pg/mL) | 3344.3 ± 5198.4 | 6790.7 ± 9364.1 | < 0.001 |
| Hb (g/dL) | 13.8 ± 2.0 | 12.8 ± 2.4 | < 0.001 |
| Na (mmol/L) | 139.4 ± 2.9 | 139.4 ± 3.4 | 0.973 |
| Cl (mmol/L) | 102.3 ± 3.7 | 103.1 ± 4.6 | 0.007 |
| K (mmol/L) | 4.4 ± 0.5 | 4.5 ± 0.6 | 0.134 |
| BUN (mg/dL) | 20.1 ± 9.3 | 27.5 ± 16.4 | < 0.001 |
| Cr (mg/dL) | 1.1 ± 0.6 | 1.8 ± 1.9 | < 0.001 |
| CCr (mL/min) | 78.9 ± 43.2 | 56.1 ± 33.0 | < 0.001 |
| Comorbidities (yes) | 414 (81.5) | 313 (90.5) | < 0.001 |
| Hypertension | 231 (45.5) | 210 (60.7) | < 0.001 |
| Atrial fibrillation | 122 (24.0) | 107 (30.9) | 0.025 |
| Dyslipidemia | 173 (34.1) | 148 (42.8) | 0.010 |
| Diabetes mellitus | 170 (33.5) | 134 (38.7) | 0.115 |
| COPD | 31 (6.1) | 26 (7.5) | 0.417 |
| MI | 66 (13.0) | 57 (16.5) | 0.155 |
| PCI | 95 (18.7) | 83 (24.0) | 0.062 |
| CABG | 18 (3.5) | 15 (4.3) | 0.556 |
| ESRD | 4 (0.8) | 28 (8.1) | < 0.001 |
| Number of comorbidities | 1.8 ± 1.3 | 2.3 ± 1.5 | < 0.001 |

Data are presented as number (%) or mean ± standard deviation.

^a^ *P* values are from Student’s independent 2-sample t-test or χ^2^ test as appropriate.

BMI, body mass index; BNP, B-type natriuretic peptide; BUN, blood urea nitrogen; CABG, coronary artery bypass grafting; CCr, creatinine clearance; Cl, chlorine; COPD, chronic obstructive pulmonary disease; Cr, creatinine; ESRD, end-stage renal disease; Hb, hemoglobin; HF, heart failure; K, potassium; LVEF, left ventricular ejection fraction; MI, myocardial infarction; Na, sodium; NT-proBNP, N-terminal pro B-type natriuretic peptide; NYHA, New York Heart Association; PCI, percutaneous coronary intervention.
